# Wildlife Rabies on an Island Free from Canine Rabies for 52 Years — Taiwan, 2013

**Published:** 2014-02-28

**Authors:** Hsiu Wu, Su-San Chang, Hsiang-Jung Tsai, Ryan M. Wallace, Sergio E. Recuenco, Jeffrey B. Doty, Neil M. Vora, Feng-Yee Chang

**Affiliations:** 1 EIS officer, CDC; 2 Taiwan Centers for Disease Control, Ministry of Health and Welfare, Taiwan; 3 Bureau of Animal and Plant Health Inspection and Quarantine, Council of Agriculture, Taiwan; 4 Animal Health Research Institute, Council of Agriculture, Taiwan; 5 Division of High-Consequence Pathogens and Pathology, National Center for Emerging and Zoonotic Infectious Diseases, CDC

Dog-to-dog transmission of rabies in Taiwan was eliminated in 1961; the island was considered canine rabies–free for 52 years. On July 16, 2013, three ferret-badgers (*Melogale moschata*) (Figure) tested positive for rabies by fluorescent antibody testing at the Animal Health Research Institute, Council of Agriculture of Taiwan. This was the first time wild animals other than bats were tested. During 1999–2012, a total of 6,841 clinically healthy dogs and five apparently normal cats from shelters were tested and found negative for rabies. During 2009–2012, a total of 322 bats were tested and found negative for rabies.

On July 23, Taiwan agriculture authorities asked forestry workers, wildlife rescue and rehabilitation stations, and local animal health agencies to submit for testing all dead wild mammals and ill wild mammals with neurologic signs, in addition to any mammals that bit or scratched humans or licked humans on broken skin or mucous membranes. Wildlife rescue and rehabilitation stations were instructed not to rehabilitate ill wild mammals exhibiting neurologic signs. Rabies vaccination campaigns for dogs and cats also were initiated.

During January 1–October 3, among samples tested at the Animal Health Research Institute, 159 of 512 (31.1%) ferret-badgers, one of 138 (0.7%) shrews, and one of 908 (0.1%) dogs tested positive for rabies. During that period, 62 cats, 44 bats, 138 wild carnivores other than ferret-badgers, and 289 other mammals also were tested and found negative for rabies. The one dog had contact with a rabid ferret-badger and developed signs of rabies while quarantined. To date, cases of rabies have been confirmed in central, southern, and eastern Taiwan; no cases have been identified in northern Taiwan.

Rural farmers and hunters who trap and slaughter ferret- badgers might be at increased risk for rabies exposure. Educational efforts are being developed for this high-risk group. To evaluate the feasibility of oral vaccination of ferret-badgers against rabies, the Council of Agriculture and CDC conducted a small-scale palatability field trial using three placebo bait types. These trials have not yet identified a bait that is universally palatable to ferret-badgers, suggesting that oral rabies vaccination of wild ferret-badgers might be difficult.

Since July 24, 2013, the Taiwan Centers for Disease Control has provided rabies vaccine and immune globulin for persons with exposure to potentially rabid animals after risk assessment using guidelines issued by Taiwan’s Advisory Committee on Immunization Practices (1,2). During July 21–October 7, a total of 4,207 persons received postexposure prophylaxis. The Taiwan Centers for Disease Control urges clinicians to maintain a high index of suspicion for rabies when evaluating patients with encephalitis in Taiwan. Surveillance for animal and human cases will need to be continued to appropriately describe the epidemiology of rabies in Taiwan.

## Figures and Tables

**FIGURE:**
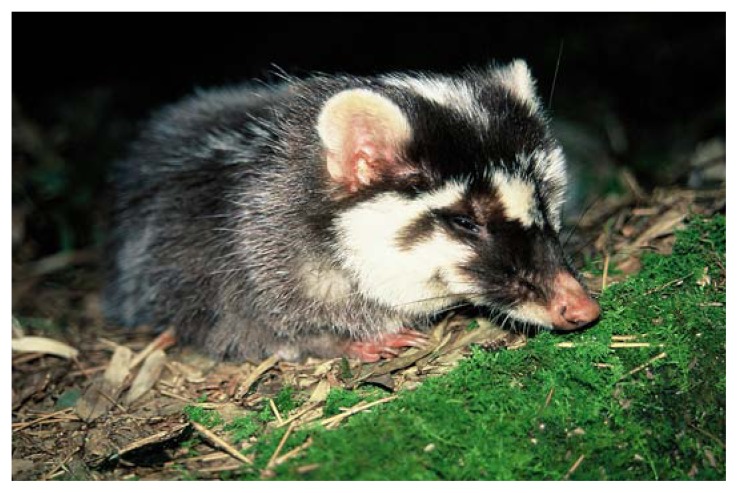
The Chinese ferret-badger, also known as the small-toothed ferret-badger (*Melogale moschata*), is widely distributed in Southeast Asia
